# Long-Term Behavior of PBO FRCM and Comparison with Other Inorganic-Matrix Composites

**DOI:** 10.3390/ma15093281

**Published:** 2022-05-03

**Authors:** Angelo Savio Calabrese, Tommaso D’Antino, Pierluigi Colombi, Carlo Poggi

**Affiliations:** Department of Architecture, Built Environment and Construction Engineering, Politecnico di Milano, 20133 Milan, Italy; angelosavio.calabrese@polimi.it (A.S.C.); pierluigi.colombi@polimi.it (P.C.); carlo.poggi@polimi.it (C.P.)

**Keywords:** FRCM, TRM, PBO, composites, tensile test, durability, long-term

## Abstract

Fabric-reinforced cementitious matrix (FRCM) composites, comprising high-strength fiber textiles embedded within inorganic matrices, represent an effective, cost-efficient, and low-invasive solution for strengthening and retrofitting existing masonry and reinforced concrete structures. Among different textiles employed in FRCM composites, polyparaphenylene benzo-bisoxazole (PBO) textiles are adopted due to their high tensile strength and good adhesion with the matrix. Although several experimental, numerical, and analytical works were performed to investigate the mechanical properties of PBO FRCM composites, limited information is available on their long-term behavior, as well as in the case of exposure to aggressive environments. This paper presents and discusses the results of a wide experimental campaign aimed at investigating the effect of different environmental conditions on the long-term tensile behavior of a PBO FRCM composite. Tests are performed using a clamping-grip tensile test set-up. The effect of various aggressive environments on the composite matrix cracking stress, composite tensile strength, ultimate strain, and fully cracked stage slope is investigated by comparing the results of nominally equal conditioned and unconditioned (control) specimens. These results are also compared with those of other FRCM composites comprising glass and carbon textiles subjected to the same conditionings, collected from the literature. The results show only limited reductions in the tensile properties, even after long exposure to aggressive environments.

## 1. Introduction

The environmental impact of the construction sector has dramatically increased over the past decades. Recently, sustainable development policies have driven growing attention toward the refurbishment of existing buildings, which has promoted the use of effective and durable strengthening materials. Among others, the use of fiber-reinforced cementitious matrix (FRCM) composites has proven to be a low-invasive and cost-efficient solution for the strengthening and rehabilitation of existing masonry and reinforced concrete (RC) structures. These materials evolved from fiber-reinforced polymer (FRP) composites by substituting the organic resin with an inorganic matrix, typically a cement- or lime-based mortar, and continuous fiber sheets with open-mesh textiles [1]. Inorganic-matrix composites have been used as externally bonded reinforcement (EBR) to increase the flexural [2,3] and shear [4,5] capacity of RC beams, as well as the axial capacity of RC columns [6,7]. They are also particularly suited for strengthening of masonry structures, where they have been employed to prevent out-of-plane [8,9] and in-plane [10,11] shear failure of masonry walls and to increase the axial capacity and ductility of masonry columns [12,13]. Indeed, the use of an inorganic matrix guarantees a better vapor permeability of the substrate with respect to that obtained with FRPs, alongside with a better behavior at high temperatures and a partial reversibility of the application [14]. Furthermore, inorganic-matrix layers of FRCM composites, being typically 5 mm thick, may provide good protection to the embedded fibers against aggressive environments, thus improving the composite durability [15].

Research on inorganic-matrix composites has been focused on the composite mechanical behavior and on the composite–substrate bond behavior. Various set-ups for tensile testing of composite coupons have been proposed. Among them, the most diffused are the clamping-grip (the coupon is directly clamped by the machine applying a certain clamping pressure [16]) and the clevis-grip set-ups (metallic plates are bonded to the coupon ends and then connected to the testing machine with clevis joints [17]). The composite–substrate bond behavior has been studied typically using single-lap shear tests due to the simplicity and clarity of results [15], while fewer studies have been carried out using double-lap shear tests [18] and beam tests [19]. 

The long-term behavior of FRCM composites is now under investigation and this will require a considerable effort from the scientific community due to the variety of textiles and matrices used in FRCM composites and to the broad range of applications, which include different substrates and several environmental conditions. Accordingly, currently available United States (US) [20] and Italian [21] acceptance criteria for FRCM composites account for specific aging/conditioning protocols and tensile test methods for the investigation of the composite long-term behavior. In addition to tensile tests on composite coupons, the European Assessment Document (EAD) [22] for FRCM includes tests aimed at assessing the composite–substrate bond durability. To account for the FRCM long-term behavior, the Italian guidelines CNR-DT 215/2018 [23] for the design of externally bonded FRCM reinforcements provide a general environmental reduction factor, which is independent from the specific type of fabric and matrix employed, while no reduction is provided by the US design guidelines ACI 549.4R-20 [24] and ACI 549.6R-20 [25] for FRCM reinforcement externally bonded to masonry and concrete members, respectively. 

In this paper, the long-term behavior of a polyparaphenylene benzo-bisoxazole (PBO) FRCM composite is experimentally investigated considering the protocols provided by the Italian acceptance criteria [21], namely hygrothermal, saline, alkaline, and dry-heat conditionings, as well as freeze–thaw cycles. Clamping-grip tensile tests of conditioned and control (unconditioned) specimens are performed to evaluate the composite performance variation after the exposure. The results obtained are compared with those of a glass and a carbon FRCM subjected to the same conditioning protocols, which are collected from the literature and presented in a state of the art.

## 2. Durability of Inorganic-Matrix Composites: A Literature Review

In this section, a literature review of the PBO FRCM long-term behavior is presented first, which includes studies at the component level, i.e., of the effect of exposures on the textile and matrix and at the system level, i.e., on the bond between the two phases and between the composite and substrate. Then, experimental results on the long-term behavior of a glass and a carbon FRCM and a steel reinforced grout (SRG) composite subjected to tensile testing are collated from the literature. For glass and carbon FRCM, only the conditioning protocols provided by the Italian acceptance criteria for FRCM composites [21], which were the same as those employed in the experimental tests of the PBO FRCM presented in this paper (see Section 3), were considered. Accordingly, results of glass and carbon FRCM collated from the literature will be compared with those of the PBO FRCM, which will shed light on the long-term tensile behavior of different inorganic-matrix composites exposed to the same conditioning protocols.

### 2.1. PBO FRCM

Due to the relevant thickness of inorganic-matrix layers constituting FRCM composites, which should be able to guarantee proper stress transfer between the fiber and substrate [19] and protect the embedded textile from the environmental exposure, the durability of PBO textiles has not been properly investigated and only a few studies are available to date. Chin et al. [26] investigated the effect of hygrothermal conditioning on the tensile strength of PBO fiber adopted for body armor equipment. The study demonstrated that the combined effect of high temperature (50–60 °C) and relative humidity (37–60% relative humidity, RH) determined a 30% reduction in tensile strength after a 26-week exposure. However, the study showed that when the same fiber was exposed to high temperature in a dry environment (5% RH) a slight reduction in tensile strength was recorded (<4%), which indicated that moisture is a key factor in the degradation of PBO fiber. In [27], the effect of alkaline environments with different chemical compositions and temperatures on the tensile strength of bare PBO textile employed in FRCM systems was investigated. The results showed that the alkalinity of lime mortar environments did not affect the PBO fiber strength even after 180 days. Tensile strength reductions of approximately 20% were observed after 90- and 180-day treatments in highly alkaline environments, namely those provided by the ETAG 029/A protocol [28] and Portland cement conditions, respectively. Higher concentrations of alkaline ions determined a 34% fiber strength reduction after a 30-day exposure. Increasing the temperature up to 45 °C accelerated the diffusion of alkaline ions in the specimens, determining 31% and 51% textile strength reductions after 60 days in lime and Portland cement environments, respectively. Ombres et al. [29] investigated the effect of high temperature on the physical and mechanical properties of a bare PBO textile for FRCM applications. Results showed that high temperatures determined a change in textile color and a significant reduction in weight (18% and 43% at 100 and 200 °C, respectively). However, the temperature did not affect the failure load of textile specimens.

FRCM composites including PBO fibers typically employ a cement-based high-performance embedding matrix due to the mechanical compatibility with the fiber. The effect of hygrothermal conditionings on the tensile and compressive strength of a short-fiber-reinforced cement-based matrix was experimentally investigated in [30] by means of compressive and bending tests. Mortar specimens were cured for 28 days in air (23 °C, 50% RH) or in water (23 °C) and then were subjected to different hygrothermal conditionings, including alternations of days of immersion and drying. Specimens showed a substantial increase in their compressive strength (34–54%) when the curing period in water was followed by an air-drying period. This was due to the fact that curing in air does not allow the specimen to reach the optimal moisturizing level of the grout. Conversely, the increase in flexural strength of specimens cured in water and then dried in air was not as significant as that observed for the compressive strength. Al-Lami et al. [31] investigated the effect of freeze–thaw cycles and saline environments on the tensile strength of cement-based matrices using results available in the literature [15,32]. These results showed that exposure to a high number of freeze–thaw cycles may reduce the matrix tensile strength due to the micro-cracks formation promoted by the volume increase of interstitial water during the freezing phase. However, this effect was not significant for a low number of cycles (<100) and was affected by the aggregate size and by the concrete strength grade, in light of the low permeability of high-strength mixtures that guarantees a low vulnerability to freeze–thaw cycles. Similarly, interstitial salt crystallization and its chemical interaction with matrix components such as calcium hydroxide and aluminum oxide determined a reduction in cement-based matrix tensile strength and durability. Results analyzed in [31] showed that this effect is enhanced by wet–dry cycles in saline environments. Finally, Ombres et al. [29] demonstrated that high temperatures affected both the compressive and flexural strength of the cement-based mortar, which decreased by 9% and 22%, respectively, at 200 °C.

The durability of both PBO fiber and cement-based matrix affects the long-term behavior of PBO FRCM composites. As previously mentioned, the external matrix layer provides protection to the textile against aggressive environments. However, premature cracking of the matrix due to shrinkage or to long-term reduction in its tensile properties may induce direct environmental exposure of the textile, which can cause fiber deterioration over time. Furthermore, exposure to aggressive environments can affect the stress-transfer mechanism between matrix and fiber, reducing the FRCM composite mechanical properties. Indeed, salt crystallization and water freezing–thawing cycles at the contact interface between textile yarns and matrix may affect the bond between the two phases, leading to potential modification of the FRCM mechanical behavior and failure mode. The currently available studies on the long-term behavior of PBO FRCM composites are based on the comparison between results of tensile tests on conditioned (control) and unconditioned composite rectangular coupons, whereas studies that consider long-term bond tests are limited [15,33]. Results obtained by Arboleda et al. [34] showed that the ultimate stress σ_u_ of PBO FRCM specimens subjected to clevis-grip tensile tests was not reduced after 1000 and 3000 h of exposure to hygrothermal, saline, and alkaline environments, twenty freeze–thaw cycles, and 4 h immersion in fuel. Conversely, conditioning protocols that accounted for immersion in aqueous solutions led to a substantial increase in the composite tensile strength, attributed to the aforementioned matrix hydration and curing, which improved the bond between the fiber yarn and surrounding matrix. The clevis-grip tensile test set-up is particularly interesting for the study of the long-term behavior of inorganic-matrix composites. Indeed, clevis-grip tensile tests allow for investigating the matrix–fiber interaction [17]. Differently, the tensile strength of conditioned specimens subjected to clamping-grip tests depends mainly on the effect of the aggressive environment on the fiber mechanical properties, since fiber rupture is the expected failure mode. The excellent long-term behavior of PBO FRCM was confirmed in [35], where 1000 h treatments were considered in saline, alkaline, hydrochloride acid, and distilled water solutions. For all the conditioning protocols, the composite exhibited a full maintenance of its control tensile strength, with some increases in the case of distilled water and acid conditionings. This suggests that when adequate preparation is provided, uncracked matrix provides adequate protection to fibers. However, the strength degradation of the composite can be significant if conditioning is performed after matrix cracking. Carozzi et al. [36] experimentally observed that when PBO FRCM specimens were pre-cracked before being subjected to freeze–thaw cycles, the corresponding ultimate stress was 36% lower than that of control specimens and 20% lower than equally conditioned non-pre-cracked specimens. In contrast with other findings, the results presented in [29] show that temperature can dramatically reduce the tensile strength of PBO FRCM composites, with 48% and 62% reductions at 100 and 200 °C, respectively. However, it should be noted that the results of control specimens are not consistent with other studies on the same material. 

The composite bond capacity with the substrate is a fundamental parameter in design practice [23] and exposure to an aggressive environment could affect it over time. The environmental temperature seems not to affect the bond capacity of PBO FRCM–concrete joints subjected to a direct shear test, as demonstrated in the experimental campaign conducted by Al-Jaber et al. [37], which considered three different testing temperatures (21, 50, and −18 °C) and high-temperature cycles (27 to 50 °C hysteresis) combined with freeze–thaw cycles. Specimens exhibited average variations in their bond capacity in the range −10% to + 19%. In [30], beam tests of PBO FRCM–concrete joints were performed on specimens conditioned in water. The results confirmed that matrix hydration in water followed by air curing improved the quality of matrix adhesion with substrate, resulting in an average of 7% bond capacity increase. Al-Lami et al. [33] investigated the effect of wet–dry cycles on the bond behavior of PBO FRCM–masonry joints subjected to direct shear tests, determining a scarce influence of the treatment on the composite bond capacity (7% reduction) after 50 cycles, which can be partially attributed to the overexposure to the wet environment of bare textile portions of the specimen.

### 2.2. Glass FRCM

Glass fiber has been increasingly used in FRCM applications due to the relatively low cost of the raw material and to the mechanical compatibility with masonry elements. However, the durability of glass fiber is significantly affected by exposure to the alkaline ions present in alkali environments. Recently, the application of a chemical zirconium treatment (alkali-resistant, AR glass) and the use of thin rubber external coating (coated glass fiber) on the fiber were proven to be effective in increasing the durability of glass fiber in alkaline environments. The long-term behavior of AR styrene butadiene rubber (SBR)-coated glass FRCM was investigated by the authors in [38] by tensile testing of rectangular coupons including one layer of a coated AR glass textile, subjected to hygrothermal, saline, alkaline, freeze–thaw cycles, and dry-heat treatments. The results showed a slight decrease in the specimen tensile strength only for saline and freeze–thaw cycles (maximum decrease equal to 12% for specimens subjected to freeze–thaw cycles). As observed for other composites, the matrix cracking stress increased after conditioning, except for specimens immersed in alkaline solutions for 1000 h, due to the continuous matrix curing. In general, the average slope *E*_3_, i.e., the slope of the fully cracked stage of the stress–strain response obtained by the clamping-grip test (see Section 4), was not affected by the exposures. *E*_3_ showed a limited decrease only for specimens subjected to the alkaline environment and freeze–thaw cycles, which was attributed to the degradation of the textile and SBR coating, respectively. Finally, the dry-heat conditioning did not induce any degradation in the tensile properties of the specimens.

### 2.3. Carbon FRCM

Carbon FRCM composites represent a promising alternative to PBO FRCM for the strengthening and retrofitting of RC elements due to the textile high elastic modulus and tensile strength. The effect of alkaline environments on a bare carbon textile for FRCM was investigated in [27]. Results showed a full retention of mechanical strength under the attack of several combinations of different types of alkaline ion. This trend was confirmed by the experimental results presented in [34], where rectangular FRCM coupons comprising a carbon textile embedded within a cement-based matrix were subjected to clevis-grip tensile tests. For all the conditionings, the carbon FRCM exhibited and increase in its ultimate stress σ_u_ and slope of the fully cracked stage, which was attributed to a continuous curing of mortar due to the immersion in aqueous solutions. In contrast, carbon FRCM specimens comprising pozzolanic matrix, subjected to a clamping-grip tensile test, presented in [39], showed significant reductions in ultimate tensile stress (40% and 33%) when subjected to alkaline and saline environments, respectively. A partial performance loss (11%) was also observed in the slope of the stress–strain curve *E*_3_ after both treatments. The effect of saline and alkaline conditionings was more pronounced if the curing time of specimens was reduced to 28 days (instead of 60) before the treatment began. For the same carbon FRCM composite, a full retention of ultimate tensile stress (113%) and elastic modulus (100%) was observed after 20 freeze–thaw cycles. In addition, the results presented in [39] shed light on the effect of environmental conditionings on the composite ultimate strain ε_u_ (i.e., strain associated with the ultimate stress σ_u_) and matrix cracking strength σ_T1_. For alkaline and saline conditioning, the ultimate strain was significantly reduced as a consequence of the aforementioned reduction in ultimate tensile stress. Conversely, the ε_u_ retained value increased after freeze–thaw cycles (113%), coherently with the increase in σ_u_. The retained matrix cracking strength, σ_T1_, was equal to 48–50% and 68–76% for 28- and 60-day cured specimens, respectively, under both saline and alkaline environments. However, it increased to 116% after the freeze–thaw cycles. The differences in the long-term behavior observed in [34,39] can be attributed to the different matrices adopted in the two composites analyzed (in the first case a cement-based matrix was employed, while a pozzolanic matrix was used in the second case) and to the different test set-ups employed (i.e., clevis-grip test in [34] and clamping-grip test in [39]). Further results are needed to clarify the effect of aggressive exposures on carbon FRCM composites.

### 2.4. Steel-Reinforced Grout (SRG)

Steel-reinforced grout systems are recently developed inorganic-matrix composites characterized by the use of continuous high-strength steel fibers as reinforcement. In SRG, steel fibers are arranged in parallel cords, realized with the wire twisting technique and held together with a polyester mesh to form a stable grid. For durability purposes, steel cords can be coated with brass or zinc to increase their corrosion resistance. Micelli et al. [27] investigated the effect of alkaline environments on the bare (i.e., not impregnated with the matrix) steel textile employed in SRG composites. The results showed a fully retained tensile strength after 30, 90, and 180 days under four different levels of alkalinity and temperature. The bare steel fiber resistance to alkaline conditioning was confirmed from the results presented in [40] on a brass-coated steel textile. In contrast, reductions in the textile tensile strength were recorded after acid conditioning (4%) and outdoor aging (20–27%). Signorini and Nobili [35] investigated the effect of different conditioning protocols on the ultimate tensile performances of two distinct SRG systems, namely a brass-coated SRG and a zinc-coated SRG, by performing clamping-grip tensile tests on rectangular coupons. The conditionings included 1000 h immersions in salt water, alkaline solution, hydrochloric acid, and distilled water. Results showed that zinc-coated SRG is more sensitive to aggressive environments than the brass-coated SRG. Indeed, the former exhibited performance reductions after all the conditionings, which were equal to 14–34% in strength and 30–60% in ultimate strain. The latter showed a 24–26% reduction in both ultimate stress and strain only in the case of hydrochloric acid conditioning, whereas performance increases were recorded after the other exposures.

## 3. Experimental Program

**Figure 1:**
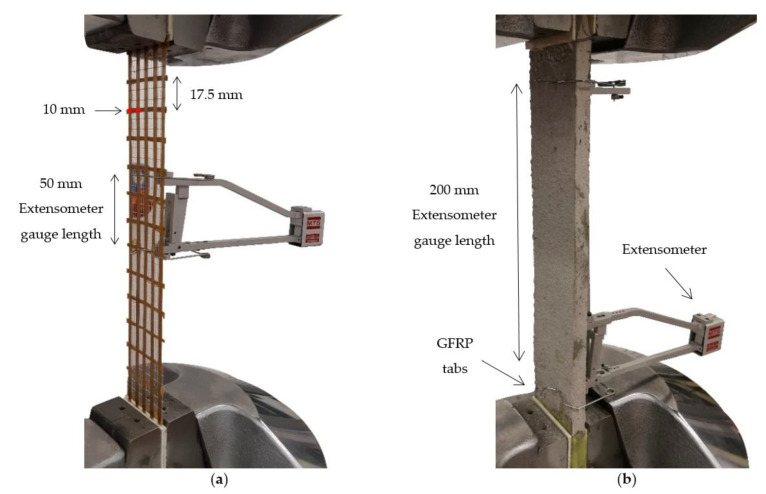
Materials characterization: (**a**) tensile test on bare textile; (**b**) clamping-grip tensile test on FRCM specimen.

The effects of five different environmental exposures on the tensile properties of a PBO FRCM composite are experimentally investigated in this study. The composite comprised an open-mesh PBO textile [41] and a cement-based mortar [42]. The textile was characterized by an unbalanced grid geometry, with longitudinal yarns spaced at 10.0 mm on center along the longitudinal (load) direction, whereas transversal yarns were spaced at 17.5 mm on center. The cross-sectional area of longitudinal yarns was equal to *A_f_* = 0.456 mm^2^. Table 1 reports the main mechanical properties of both textile and matrix, namely the tensile strength σ_fu_, the associated ultimate strain ε_fu_, and the elastic modulus *E*_f_ of the textile in the longitudinal direction. Furthermore, Table 1 reports the matrix tensile strength σ_mu_, the compressive strength *f_c_*, the flexural strength *f_r_*, and the elastic modulus *E*_m_. The tensile properties of the textile were obtained by tensile testing of nine 50 mm wide by 400 mm long bare (i.e., not impregnated) textile strips including 5 longitudinal yarns according to [22] (see Figure 1a), whereas the compressive and flexural strengths of the matrix were experimentally obtained according to UNI EN 1015-11 [43]. These results have already been reported in a previous paper by the authors [44]. The tensile strength and elastic modulus of the matrix were taken from [17].

The environmental conditionings considered in this study were conducted according to the indications of the Italian acceptance criteria for FRCM composites [21] and included hygrothermal, saline, alkaline, freeze–thaw cycles, and dry-heat conditionings. Forty-five FRCM coupons of dimensions 400 mm (length) × 50 mm (width) × 10 mm (thickness), including one layer of PBO textile (overall fiber cross-sectional area *A* = 5 × *A_f_* = 2.28 mm^2^) embedded within two 5 mm-thick matrix layers were realized according to the indications of [21] and subdivided into 9 groups of five specimens each. One group was unconditioned and adopted as control, two groups were subjected to the hygrothermal conditioning for 1000 and 3000 h, respectively, and the same was done for the saline and alkaline conditionings. The remaining two groups of specimens were subjected to freeze–thaw cycles and dry-heat conditionings, respectively. 

Specimens were named according to the notation T_400_50_XC_n, where T (= tensile) is the test method, 400 and 50 are the specimen length and width in mm, respectively, XC indicates the conditioning method, where X (if present) refers to the exposure duration (X = 1 stands for 1000 h and X = 3 for 3000 h), C is the environmental exposure type (NC = control, W = hygrothermal, S = saline, A = alkaline, F/T = freeze–thaw cycles, T = dry-heat), and n is the specimen number.

The five conditioning protocols considered in this study were performed as follows:

- Hygrothermal: specimens were stored in an environmental chamber at 92% RH and 38 °C for 1000 h and 3000 h.
- Saline: specimens were immersed in a saline solution [45] at 23 ± 2 °C for 1000 h and 3000 h.
- Alkaline: specimens were immersed in an alkaline solution having 9.5 pH, at a temperature of 23 ± 2 °C for 1000 h and 3000 h.
- Freeze–thaw cycles: specimens were stored in an environmental chamber for 7 days at 92% RH and 38 °C and then were subjected to 20 freeze–thaw cycles, each one consisting of 4 h at −18 °C followed by 12 h at 92% RH and 38 °C.

Dry-heat: specimens were stored in an environmental chamber where the temperature was increased at a rate of 15 °C every 30 min until reaching 100 °C. Then, the temperature was maintained for 6 h. Specimens were taken from the chamber and tested in a thermal cell where the temperature was maintained at 100 °C during the entire test.

Figure 1b shows the clamping-grip tensile test configuration adopted for the PBO FRCM specimens. Tests were conducted in displacement control at a stroke rate of 0.2 mm/min during the uncracked phase and of 0.5 mm/min during the cracked phase, according to the indications of [21]. A servo-hydraulic testing machine with hydraulic wedge grips was employed for the test. The machine wedge pressure was set to provide adequate gripping for the specimen and to prevent textile slippage within the clamped length. The coupon ends were equipped with GFRP tabs with dimensions 80 mm × 50 mm × 2 mm to promote an even distribution of the clamping pressure and prevent mortar failure within the wedges. As a result, the specimen free length, i.e., excluding the clamped portions, was equal to L_0_ = 240 mm. A 200 mm-gauge length extensometer was adopted for the strain reading along the central portion of the specimen (Figure 1b).

## 4. Results

**Figure 2:**
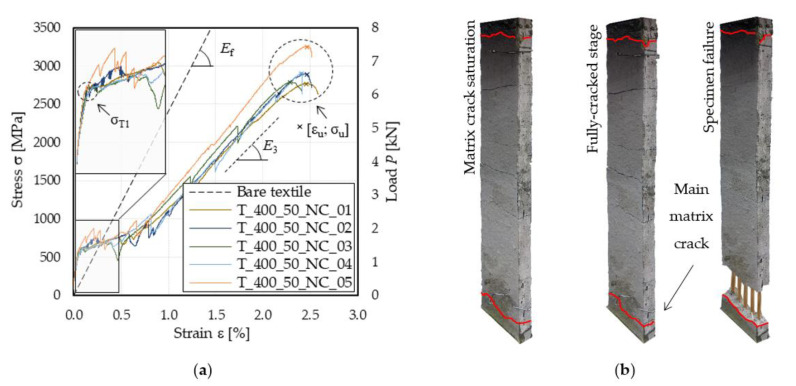
Clamping-grip tensile tests: (**a**) stress (load)–strain curves; (**b**) test of specimen T_400_50_NC_04: matrix cracks outside the extensometer gauge length are marked in red.

Figure 2a shows the stress σ–strain ε response of unconditioned FRCM specimens, where σ = *P*/*A* is the ratio between the load applied by the testing machine, *P*, and the fiber overall cross-sectional area, *A*. The specimen stress–strain responses showed an almost trilinear behavior [16], with an initial linear branch associated with the specimen uncracked stage. In this stage, the specimen axial behavior was governed mainly by the matrix mechanical properties, until the matrix cracking stress, σ_T1_ (Figure 2a), was reached. Beyond this point, cracks opened along the specimen length, determining stress drops in the load response while the tensile stress was gradually transferred from the matrix to the textile until the full matrix crack saturation was attained and the fully cracked stage began (Figure 2b). During this final stage, cracks previously opened became wider with the increase in the applied displacement and the behavior of the specimen was mainly governed by the textile properties. The specimen failure occurred for all specimens due to fiber rupture within a matrix crack, as shown in Figure 2b, at a stress value σ_u_ corresponding to the axial strain value ε_u_ (Figure 2a). Table 2 reports the main parameters of the stress (load)–strain response of unconditioned specimens, where the matrix cracking stress, σ_T1_, was identified in correspondence to the first stress (load) drop of the response (Figure 2a) and the stress–strain response slope during the fully cracked stage, *E*_3_, was computed by linear regression of the points within 0.6 σ_u_ and 0.9 σ_u_ [20].

As inferred from Figure 2b, during the crack opening stage, some matrix cracks occurred outside the extensometer gauge length (marked in red in Figure 2b). Accordingly, the elongation measured by the instrument may not be representative of the entire specimen [17], particularly if the main matrix crack (i.e., the one where fiber failure is located) occurs close to the grips. For this reason, in this paper the strain ε was computed as the ratio between the machine stroke displacement reading (δ) and the specimen free length (L_0_), i.e., ε = δ/L_0_.

Unconditioned specimens showed an average matrix cracking stress ${\bar{\sigma }}_{T1}$ = 617 MPa, which corresponded to an axial load of 1.40 kN and a matrix tensile stress of 2.80 MPa, considering a nominal matrix area of 500 mm^2^. This cracking stress was 25% lower than the mortar tensile strength (σ_mu_ = 3.75 MPa, see Table 1). This can be attributed to differences between the actual and nominal cross-sectional area considered and to stress concentration localized at the end of the gripped length of the specimen, due to the clamping pressure applied by the testing machine [17]. Similarly, the average ultimate stress of specimens ${\bar{\sigma }}_{u}$ was 26% lower than the bare textile tensile strength σ_fu_ (Table 1), which indicates that in the majority of the specimens the applied load was unevenly distributed among the fiber yarns, resulting in stress concentrations in individual fiber filaments. The specimens average fully cracked stage slope ${\bar{E}}_{3}$ was 40% lower than that of bare textile strips (Table 1). This remarkable difference is attributed to the different methods adopted for measuring the specimen axial strain (extensometer for the bare textile and machine stroke for the FRCM) .

### Behavior under Different Environmental Conditions

The effect of environmental exposures on the mechanical properties of the PBO FRCM analyzed in this study was evaluated by comparing the average mechanical properties obtained by control specimens, shown in Table 2, with those of conditioned specimens. 

**Figure 3:**
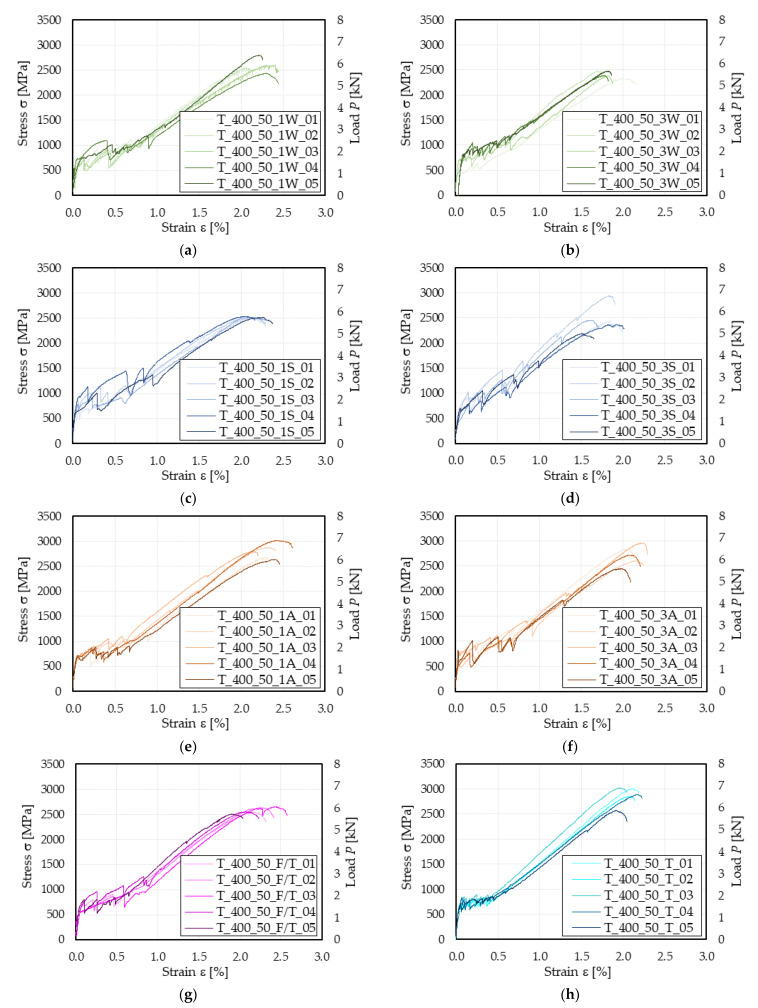
Results of conditioned FRCM coupons: (**a**) hygrothermal, 1000 h; (**b**) hygrothermal, 3000 h; (**c**) saline, 1000 h; (**d**) saline, 3000 h; (**e**) alkaline, 1000 h; (**f**) alkaline, 3000 h; (**g**) freeze–thaw cycles; (**h**) dry-heat (100 °C).

For all conditioned specimens, failure occurred due to textile rupture following matrix cracking. The corresponding σ–ε responses are grouped per conditioning type and reported in Figure 3. All tested specimens exhibited a trilinear stress–strain response, which was consistent with that of control specimens (see Figure 2a). Table 3 reports the average values of the matrix cracking stress, ultimate tensile stress, associated ultimate strain, and fully cracked stage slope obtained by each group of conditioned specimens, along with the corresponding coefficient of variation and percentage of retained performance (Ret. %), computed as the ratio between the average result of conditioned specimens and that of control specimens.

The hygrothermal conditioning determined a 13% and 18% average ultimate stress reduction at 1000 h and 3000 h, respectively. A similar trend was recorded for the average ultimate strain ${\bar{\varepsilon }}_{u}$ reduction, which was 10% and 24% after 1000 and 3000 h, respectively. This behavior can be attributed to the detrimental effect of humidity and temperature on the PBO fiber [26], which was only partially prevented by the matrix protection. The exposure to a high-RH environment determined a continuous hydration and curing of the cement-based matrix, which determined a slight increase in its tensile properties, reflected by the 7% increase in average matrix cracking strength measured after 3000 h. The fully cracked stage slope of specimens subjected to hygrothermal conditioning was on average 18% and 9% lower than that of control specimens. The higher retained percentage measured after 3000 h can be explained by the increase in matrix performances, which could have improved the quality of the matrix–fiber interface.

Specimens subjected to saline environment conditioning exhibited a 14–15% reduction in their average tensile strength after both 1000 and 3000 h. The ultimate tensile strain of specimens conditioned for 3000 h was on average 18% lower than that of specimens conditioned for 1000 h and in both cases performance losses were recorded (−11% at 1000 h, −27% at 3000 h). Since studies investigating the effect of salt on PBO fibers are not available in the literature, these performance reductions may be attributed to the high-moisture conditions of the textile during the treatment. The average fully cracked stage slope ${\bar{E}}_{3}$ of 1000 h conditioned specimens decreased by 24%, while that of 3000 h conditioned specimens by 14%. The immersion in salt water determined a continuous hydration of the matrix, which entailed a 15% increase in ${\bar{\sigma }}_{T1}$ after 1000 h of treatment and an almost full retention of it after 3000 h (97 Ret. %, see Table 3). However, it should be noted that the scatter of matrix cracking strength values is significantly high (see CoV values in Table 3), which can be attributed to irregularities in specimen geometry, presence of air bubbles, and shrinkage cracks.

The conditioning in alkaline solution determined a slight decrease (4%) in the specimens average ultimate stress and strain after 1000 h of treatment. The reduction in and ${\bar{\varepsilon }}_{u}$ was more pronounced after 3000 h, with reductions of 7% and 13%, respectively. This finding is in line with the results presented by Micelli et al. [27], which indicated a certain sensitivity of the PBO fiber to the alkali attack, which was not adequately prevented by the matrix. ${\bar{E}}_{3}$ was reduced by 16% and 13% after 1000 h and 3000 h, whereas ${\bar{\sigma }}_{T1}$ remained almost unaltered for both durations (102% and 103% retained values, respectively). 

Specimens subjected to freeze–thaw cycles exhibited a 12% reduction in their average tensile strength. In contrast, their average ultimate strain was almost fully retained (98 Ret. %), which in turn reflected a slightly lower (6%) fully cracked stage slope compared with that of control specimens. Remarkably, the matrix properties were significantly affected by the conditioning and this was the only case of unretained average matrix cracking strength (77%). This can be attributed to the formation of micro-cracks due to the volume increase in pore water during the freezing stage. However, it should be noted that a high scatter affected the evaluation of ${\bar{\sigma }}_{T1}$ (CoV = 26.83%, see Table 3) and further studies are needed to clarify if the decrease in strength should be attributed to the matrix randomly distributed properties or to the conditioning.

Specimens exposed to dry-heat conditioning at the maximum service temperature of 100 °C for 6 h maintained their performances for all mechanical parameters considered in this study (−4% ≤ Ret. % ≤ 3%). This represents an advantage of FRCM composites compared to FRP composites, for which a similar temperature would have altered the polymeric resin mechanical and physical properties [46]. 

## 5. Comparison between the Long-Term Behavior of Different FRCM Systems 

**Figure 4:**
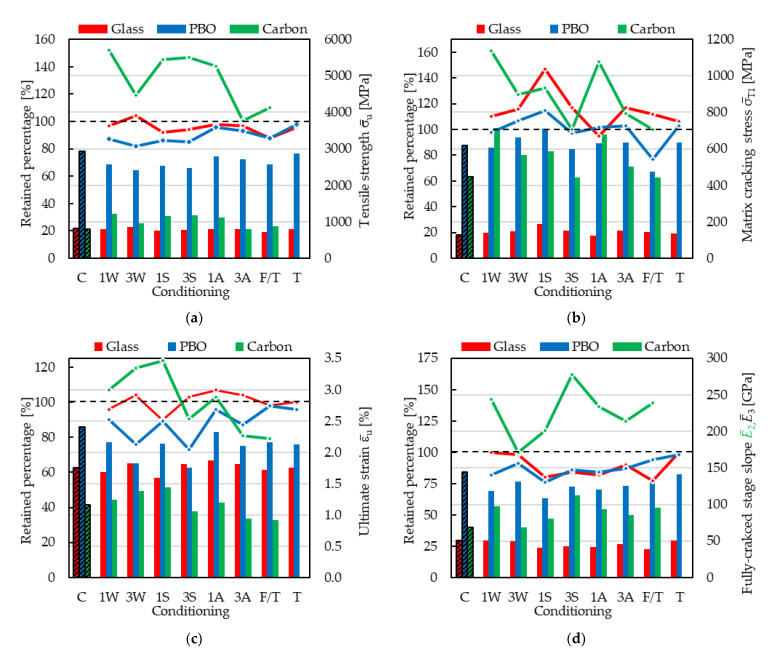
Average mechanical properties of FRCM composites after different environmental conditionings (histogram bars) and corresponding percentages of retained performances with respect to control specimens (line charts). (**a**) Tensile strength; (**b**) matrix cracking strength; (**c**) ultimate strain; (**d**) slope of the fully cracked stage. Nomenclature: C = Control specimens (unconditioned), W = Hygrothermal, S = Saline, A = Alkaline, F/T = Freeze–thaw cycles, T = maximum service temperature.

In this section, the results obtained from the PBO FRCM are compared with those of a carbon and an alkali-resistant (AR) glass FRCM reported in Sections 2.1 and 2.3, respectively. All composites considered were subjected to the same conditioning protocols described in Section 3. The results of SRG provided in Section 2.4 are not included in this comparison since they were obtained with different conditionings. Figure 4 compares the average tensile strength (Figure 4a), matrix cracking stress (Figure 4b), ultimate strain (Figure 4c), and slope of the fully cracked stage (Figure 4d) after the exposures (histogram bars) and the corresponding percentages of retained performances with respect to control specimens (line charts). Average values were computed from groups of five specimens per conditioning and are reported in Table 4 and Table 5 for carbon [34,47] and AR glass [38] FRCMs. Values provided for the PBO FRCM were those presented in this paper. It should be noted that carbon, AR glass, and PBO FRCMs employed a cement-based matrix. However, tensile tests on PBO and AR glass FRCMs were performed using the clamping-grip set-up [21], whereas the clevis-grip set-up was employed for the carbon FRCM [20]. Accordingly, the average slope of the fully cracked stage of carbon FRCM specimens, which is named cracked elastic modulus in ACI 549 [24,25], is indicated with the symbol ${\bar{E}}_{2}$ in Figure 4d and Table 4. Furthermore, carbon FRCM specimens were not exposed to dry-heat conditions (column T = maximum service temperature in Figure 4).

Carbon FRCM exhibited the best long-term performances among the three composites considered, reporting no reductions in the composite average ultimate stress or in its average cracked elastic modulus (${\bar{E}}_{2}$). This trend, and particularly the relevant increases recorded in retained values of average parameters (up to 162%), can be attributed to the test set-up adopted for the tests (clevis-grip), which was able to capture the improvement of matrix–fiber interface capacity resulting from the continuous curing and hydration of the cement-based matrix during the conditionings, shown by the increase in matrix average cracking stress after all conditionings (see Figure 4b). Both PBO and AR glass FRCMs were subjected to the same clamping-grip tensile test set-up and the latter showed a slightly better long-term behavior, with performance reductions ranging from 2% to 12% in the composite tensile strength and from 2% to 23% in its fully cracked stage slope ${\bar{E}}_{3}$ (see Table 5).

The scatter in the composite ultimate strain (ε_u_) observed for all systems was influenced by the variations in both σ_u_ and *E*_3_ (or *E*_2_ for carbon). In this regard, it should be noted that values of ${\bar{\varepsilon }}_{u}$ of carbon FRCM reported in Table 4 were computed as the ratio between ${\bar{\sigma }}_{u}$ and ${\bar{E}}_{2}$, since they were not reported in [34,47]. For this reason, the CoV associated with ${\bar{\varepsilon }}_{u}$ is not reported in Table 3. 

Figure 4b reports a general increase in the matrix cracking stress after all treatments, which indicates a poor detrimental effect of those aggressive environments on the cement-based matrix properties. The freeze–thaw cycles were the only exception to this trend.

## 6. Conclusions

In this paper, the long-term behavior of a PBO FRCM composite was experimentally investigated. Clamping-grip tensile tests on conditioned and control specimens were performed. Different exposure conditions, namely hygrothermal, saline, alkaline, and dry-heat conditionings, as well as freeze–thaw cycles, were considered. The conditioning protocols were those provided by the Italian acceptance criteria for FRCM composites. The results obtained were discussed and compared with those of a glass and a carbon FRCM subjected to the same conditioning protocols. Results of the PBO FRCM composite investigated in this paper showed a general limited decrease in the average tensile strength after exposure, with the highest decreases in the case of specimens exposed to hygrothermal conditions for 3000 h (i.e., 18% on average) and to a saline environment (i.e., 14% and 15% on average after 1000 h and 3000 h, respectively). However, the matrix average cracking strength slightly increased with conditioning (i.e., 102–115% retained percentages) due to the continuous curing of the matrix, except for specimens subjected to freeze–thaw cycles, which exhibited a 23% ${\bar{\sigma }}_{T1}$ reduction. Comparison of these results with those of a glass and a carbon FRCM showed that the carbon FRCM was the least affected composite in terms of ultimate tensile stress, while mixed results were obtained in terms of matrix cracking stress, composite ultimate strain, and slope of the stress–strain curve fully cracked stage. 

The results obtained shed light on the long-term behavior of different inorganic-matrix composites subjected to the same conditioning protocols. These results generally confirm the good durability of inorganic-matrix composites. However, the variability of results obtained points out the need for defining specific environmental coefficients to account for the long-term behavior of each composite type. 

## Figures and Tables

**Table 1:** Mechanical properties of fiber textile and matrix.

	PBO Warp Textile (CoV)	Matrix (CoV)
Tensile strength—σ_fu_, σ_mu_	3960 MPa ^†^ (0.07)	3.75 MPa ^¤^
Elastic modulus—*E*_f_, *E*_m_	241 GPa ^†^ (0.05)	7.0 GPa ^¤^
Tensile strain—ε_fu_	1.65 % ^†^ (0.08)	-
Compressive strength *f_c_*	-	51.60 MPa ^†^ (0.03)
Flexural strength *f_r_*	-	8.10 MPa ^†^ (0.16)

^†^ Taken from [44]. ^¤^ Taken from [17].

**Table 2:** Results of tensile tests of control PBO FRCM coupons.

Specimen	σ_T1_ [MPa]	*P*_u_ [kN]	σ_u_ [MPa]	ε_u_ [%]	*E*_3_ [GPa]
T_400_50_NC_01	621	6.321	2778	2.46	126
T_400_50_NC_02	628	6.600	2901	2.45	132
T_400_50_NC_03	613	6.364	2797	2.29	145
T_400_50_NC_04	609	6.626	2913	2.41	162
T_400_50_NC_05	613	7.450	3275	2.46	155
Average	617	6.672	2933	2.41	144
CoV	1.11%	6.11%		2.64%	9.40%

**Table 3:** Average results of conditioned specimen groups and retained performance.

Specimens	${\bar{\sigma }}_{T1}\;$		${\bar{P}}_{u}$	${\bar{\sigma }}_{u}$		${\bar{\varepsilon }}_{u}\;$		${\bar{E}}_{3}\;$	
	[MPa]	Ret. %	[kN]	[MPa]	Ret. %	[%]	Ret. %	[GPa]	Ret. %
	(CoV)		(CoV)			(CoV)		(CoV)	
T_400_50_1W	607	98%	5.835	2565	87%	2.16	90%	118	82%
	(8.26%)		(5.45%)			(6.89%)		(7.40%)	
T_400_50_3W	662	107%	5.496	2416	82%	1.82	76%	131	91%
	(24.69%)		(2.35%)			(5.37%)		(11.88%)	
T_400_50_1S	711	115%	5.749	2527	86%	2.14	89%	109	76%
	(14.69%)		(0.29%)			(3.32%)		(9.09%)	
T_400_50_3S	599	97%	5.647	2482	85%	1.75	73%	124	86%
	(21.00%)		(10.06%)			(9.25%)		(10.04%)	
T_400_50_1A	630	102%	6.377	2803	96%	2.32	96%	120	84%
	(8.69%)		(4.75%)			(4.05%)		(3.75%)	
T_400_50_3A	634	103%	6.184	2718	93%	2.10	87%	125	87%
	(22.79%)		(6.40%)			(4.20%)		(8.67%)	
T_400_50_F/T	476	77%	5.870	2580	88%	2.16	98%	135	94%
	(26.83%)		(1.96%)			(8.13%)		(7.74%)	
T_400_50_T	634	103%	6.563	2873	98%	2.13	96%	141	98%
	(5.69%)		(5.35%)			(3.49)		(5.60%)	

**Table 4:** Carbon FRCM: average results of conditioned specimen groups and retained values.

**Conditioning**		${\bar{\sigma }}_{T1}\;$		${\bar{P}}_{u}$	${\bar{\sigma }}_{u}$		${\bar{\varepsilon }}_{u}\;$		${\bar{E}}_{2}\;$	
	**Ref.**	[MPa]	Ret. %	[kN]	[MPa]	Ret. %	[%]	Ret. %	[GPa]	Ret. %
		(CoV)		(CoV)			(CoV)		(CoV)	
Unconditioned (C)	[34,47]	445	-	1.922	802	-	1.16	-	69	-
		(34%)		(4%)			(-)		(19%)	
Hygrothermal 1000 h (1W)		716	161%	2.922	1219	152%	1.24	107%	98	142%
		(23%)		(16%)			(-)		(19%)	
Hygrothermal 3000 h (3W)		565	127%	2.294	957	119%	1.39	119%	69	100%
		(25%)		(14%)			(-)		(19%)	
Saline 1000 h (1S)		588	132%	2.793	1165	145%	1.44	124%	81	117%
		(41%)		(18%)			(-)		(18%)	
Saline 3000 h (3S)		444	100%	2.826	1179	147%	1.05	91%	112	162%
		(33%)		(13%)			(-)		(32%)	
Alkaline 1000 h (1A)		678	152%	2.699	1126	140%	1.20	103%	94	136%
		(30%)		(12%)			(-)		(29%)	
Alkaline 3000 h (3A)		501	113%	1.937	808	101%	0.94	81%	96	139%
		(20%)		(8%)			(-)		(23%)	
Freeze–thawcycles (F/T)		444	100%	2.117	883	110%	0.92	79%	96	139%
		(36%)		(9%)			(-)		(23%)	
Dry-heat (T)		-		-			-		-	

**Table 5:** Glass FRCM: average results of conditioned specimen groups and retained values.

**Conditioning**		${\bar{\sigma }}_{T1}\;$		${\bar{P}}_{u}$	${\bar{\sigma }}_{u}$		${\bar{\varepsilon }}_{u}\;$		${\bar{E}}_{3}\;$	
	**Ref.**	[MPa]	Ret. %	[kN]	[MPa]	Ret. %	[%]	Ret. %	[GPa]	Ret. %
		(CoV)		(CoV)			(CoV)		(CoV)	
Unconditioned (C)	[38]	128	-	2.608	828	-	1.75	-	51	-
		(16.21%)		(6.62%)			(7.87%)		(3.38%)	
Hygrothermal 1000 h (1W)		141	110%	2.523	801	97%	1.69	97%	51	100%
		(26.30%)		(5.50%)			(5.90%)		(2.70%)	
Hygrothermal 3000 h (3W)		149	116%	2.706	859	104%	1.82	104%	50	98%
		(22.00%)		(4.60%)			(3.80%)		(1.90%)	
Saline 1000 h (1S)		188	147%	2.388	758	92%	1.59	91%	41	80%
		(15.50%)		(13.50%)			(14.00%)		(16.70%)	
Saline 3000 h (3S)		150	117%	2.441	775	94%	1.81	103%	43	84%
		(8.30%)		(12.90%)			(15.00%)		(11.50%)	
Alkaline 1000 h (1A)		122	95%	2.545	808	98%	1.87	107%	42	82%
		(15.10%)		(5.00%)			(5.20%)		(17.00%)	
Alkaline 3000 h (3A)		150	117%	2.529	803	97%	1.81	103%	46	90%
		(6.60%)		(6.30%)			(5.00%)		(8.60%)	
Freeze–thawcycles (F/T)		143	112%	2.284	725	88%	1.72	98%	39	77%
		(12.80%)		(10.30%)			(7.10%)		(6.20%)	
Dry-heat (T)		136	106%	2.492	791	96%	1.75	100%	51	100%
		(28.40%)		(4.10%)			(5.60%)		(2.90%)	

## Data Availability

The data presented in this study are available on request from the corresponding author. The data are not publicly available because the research is still ongoing.
